# Design and fabrication of a super-wideband transparent antenna implanted on a solar cell substrate

**DOI:** 10.1038/s41598-023-37073-5

**Published:** 2023-06-20

**Authors:** Mohammadreza Rohaninezhad, Meysam Jalali Asadabadi, Changiz Ghobadi, Javad Nourinia

**Affiliations:** Department of Electrical Engineering, Urmia University, 57153 Urmia, Iran

**Keywords:** Electrical and electronic engineering, Magnetospheric physics

## Abstract

In this study, an antenna with transparent super wideband CPW technology has been designed and built with the combination of solar panels for use in wireless communication equipment and systems that require mobile power. The transparency value of the antenna is 63.3%, which is an acceptable value for the optimal use of sunlight. The proposed antenna was designed and measured on a plexi-glass substrate with a dielectric constant of ${{\varvec{\upvarepsilon}}}_{{\varvec{r}}}=4.4$ and various thicknesses, the proposed antenna was designed and measured. The copper sheet has a high electrical conductivity and therefore it was selected for the antenna's radiating component as compared to earlier techniques that employed metal oxide. All simulations were done with CST microwave studio software and frequency domain solver. The results indicated that the antenna's operating frequency ranges from 2 to 32 GHz. The results of the computations indicated that the antenna's peak gain and peak efficiency are 8.1 dB and 90%, respectively. Various multiple-input and multiple-output (MIMO) performance parameters, including the envelope correlation coefficient (ECC), diversity gain (DG), average effective gain (MEG), total active reflection coefficient (TARC), and channel capacity loss (CCL), were analyzed to demonstrate the antenna's performance.

## Introduction

Microstrip Antenna has recently attracted attention, due to its unique characteristics such as including its wide width, tiny size, straightforward construction, and ease of adaption. A range of 3.1–10.6 GHz was allocated to such means by (FCC) which is now familiar to ultra wideband (UWB) designers^1^. Radio technology using the ultra wideband (UWB) has the ability to transfer data faster across small distances. UWB antennas are helpful for biomedical applications like imaging lung cancer^2–5^. Recently the use of SWB frequency range has received attention to the point that can cover and provide short and long-range data transmitting services^6–8^.

Super wideband (SWB) technology can be utilized for rapid sound and video transfer since it has a broad bandwidth and a large range of data capacity. Currently, this technology is employed for controlling and supervising purposes in both the private and military sectors. SWB in comparison to UWB has a bigger channel capacity, better timing accuracy, and higher image resolution^9^. Furthermore, short-long-range transmission systems and the new generation of telecommunications can employ antennas with a broader bandwidth^10^.

The nominal bandwidth, which is the difference between Higher frequency (${f}_{H}$) and lower frequency (${f}_{L}$) in 10 dB, can be used to describe antenna bandwidth with regard to the percentage of the bandwidth. We can calculate the bandwidth as follows:

1 $$BR=\frac{BW}{{f}_{L}}$$

2 $$R=\frac{{f}_{H}}{{f}_{L}}$$

3 $$BR=R:1$$

Here $BW$ fractional bandwidth, ${f}_{L}$ is the lower frequency of the operation band and ${f}_{H}$ is the higher frequency of the operation band. This ratio in SWB should be 10:1 or more, which is a desirable bandwidth^11^. UWB antennas have been the subject of extensive research. Ultra-wideband (UWB) communications provide a large bandwidth with a high data rate, low transmission power with lengthened battery life and secure communications, and short pulse time modulation with reduced multipath fading. Also, UWB technology has many applications such as localization, imaging, radars, wireless body sensor networks, and short-range large bandwidth communications^12–15^. These works have focused on the microscopic SWB geometry and its use in contemporary communications. For example, In^16^ personal wireless UWB monopole antenna was designed for the Bandwidth of 135.2%, and the usage of 79.21%. In^17^ the author designed a practical antenna that may be utilized for WIMAX/WLAN/ISM and other wireless applications with a bandwidth of 153.22% and a bandwidth fraction of 86%. In^18^, a monopole antenna with a Bandwidth of 3.1–10.6 GHz and 82.22% is the efficiency of the higher band has been designed for wireless. In^19^, a UWB antenna with two circles of connected lines was designed in a frequency Band of 3.85–0.55 GHz with a 90.1% efficiencies. Finally, in^20^ a monopole antenna with a zigzag shape for an access to 102% is designed to be employed in communication processes.

An antenna with line (TL) loaded, compact, ultrawideband (UWB) square slot antenna is a radiation efficiency of 79.21% has also been designed for personal wireless communication systems. The square slot is a microstrip line fed and loaded with an array of periodically perturbed TLs to achieve a UWB response from 2.1 to 11.5 GHz^21^. A slotted waveguide antenna with a radiation rate of 88% was presented. A slotted waveguide antenna with a radiation rate of 88% was presented. The proposed antenna has a small size of 30 mm × 35 mm, a simple geometry, and the excitation is launched through a 50 Ω microstrip feed line^22^. The use of fractal geometry in antenna design provides a good method for achieving the desired miniaturization and multi-band properties. In this communication, a multi-band and broad-band microstrip antenna based on a new fractal geometry is presented. The proposed design is an octagonal fractal microstrip patch antenna. The results show that the proposed microstrip antenna can be used for 10–50 GHz frequency range, i.e., it is a super wideband microstrip antenna with 40 GHz bandwidth^23^. All of these antennas employ a monopole micro strip that has a UWB bandwidth of 40 GHz (50–10 GHz), which can easily be made by precisely constructing a microwave and solar cell combination that is useful for satellite and ground applications. For ground purposes, it has led to expanding the automatic integrated system in driver-less cars^24^.

Recently a significant volume of research has been done in designing solar antennas with smaller sizes and the capability of producing DC voltage while transferring microwave signals. As an example we refer to the microstrip transparent antenna in^25^ This antenna was made of an expensive luminous conductor film AGHT-4 with a minimum visible light transfer of 75%. The suggested antenna cannot be utilized in multi-band or broadband communication systems since it is only designed for a single frequency band with an impedance bandwidth of 4.3%. In^26^, a super-wideband antenna based on a propeller shaped printed monopole with CPW feed is presented. The enhanced bandwidth is obtained by modifying the disk of a conventional circular disk monopole to resemble a propeller. This design produces an extremely wide impedance bandwidth from 3 to 35 GHz with an impedance bandwidth ratio of 11.6:1. The gain of the proposed antenna varies from 4 to 5.2 dB. In^27^, a MIMO antenna with a circle ring of coating of silver-tin alloy radiation patch over a layer of plexi-glass is recommended. This antenna features a 3.7 GHz WLAN and a 2.4 GHz WIMAX. The employment of silver-tin-alloy as a radiator has a negative effect on radiation performance, in addition to the high cost and complexity of production, and the researchers have proposed a solution in the form of MIMO in this study to solve this issue. In^28^, a printing technique on glass is used. the mask-covered glass uniformly with the photolithography technique. Finally, due to the thickness the of the glass, no negative effects happened to the Pyrex glass while printing ink of silver. The practical accuracy of fabrication is 0.02 mm for the mask and etching mechanism. the metallic pattern screen is plated with silver on the glass.

In this paper, we present a transparent super wide band antenna with solar-cells. At the first step we designed the antenna on a FR4 substrate. Next, transparent plexi-glass was used as a transparent substrate. In previous works, metal oxides is used for the construction of radiation part, which can interfere with the antenna radiation function. In our case, a layer of copper sheet that is precisely cut by CNC laser is used. This antenna can be used in satellite with its solar cells. It can also be utilized in vehicles glasses, wireless CCTV cameras and wherever there is a need for electrical power supply beside antenna. We will discuss this in the future.

## Antenna design

### Designing antenna on FR4 substrate

**Figure 1:**
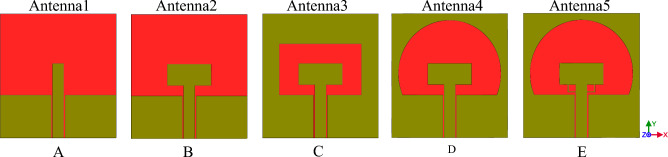
The designing processes of the SWB antenna, respectively from (**A**) to (**E**) are depicted.

We design this antenna with CPW feeding Technology. As shown in Fig. 3, a transfer line CPW with W (central line Wideband) parameter, g (the distance from the same ground level) and h (angle altitude dielectric) are depicted. The distance g should be less than W, otherwise the necessary coupling between the mild line and S surrounded ground plane will not be achieved. The calculation for the transfer line is done with AWR microwave software office and TX-line tools. At the first stage, we have designed the antenna over the FR4 substrate with $\mu =1$, ${\varepsilon }_{r}=4.3$ and thickness of 1.6 mm. In Fig. 1, the designing steps are shown.

**Figure 2:**
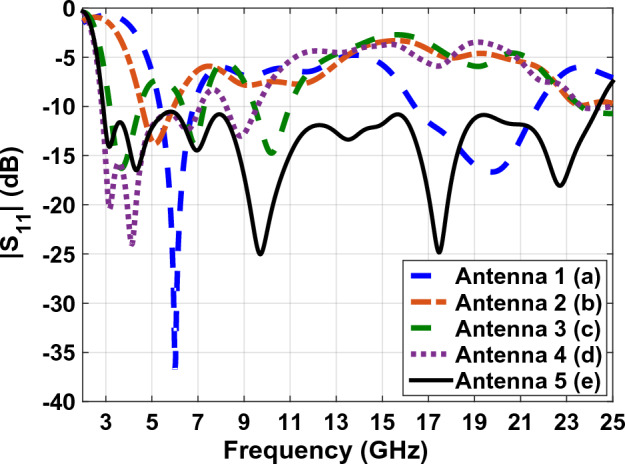
The comparing graphs of antennas in Fig. 1.

As shown in Fig. 1 in the first step, we have designed antenna1, which is made of CPW line without radiation patch the operating frequency range of this antenna varies from 5.3 to 6.8 GHz and it is measured from 16.4 to 21.7 GHz. This frequency range cannot be used for wide Band purposes. In the second step (Fig. 1B) the frequency is decreased by adding the primary radiation patch 10.2 × 5 mm to the feeding line. As shown in Fig. 2 scattering parameter of the antenna is only in Bandwidths 4.54–5.95 GHz under 10 dB. That is, the variation in 1B will cause a better impedance in lower frequency with respect to the antenna in Fig. 1A. However, our goal is to build a 3.1 GHz antenna according to the Federal Communications Commission (FCC) for wideband programs.

**Figure 3:**
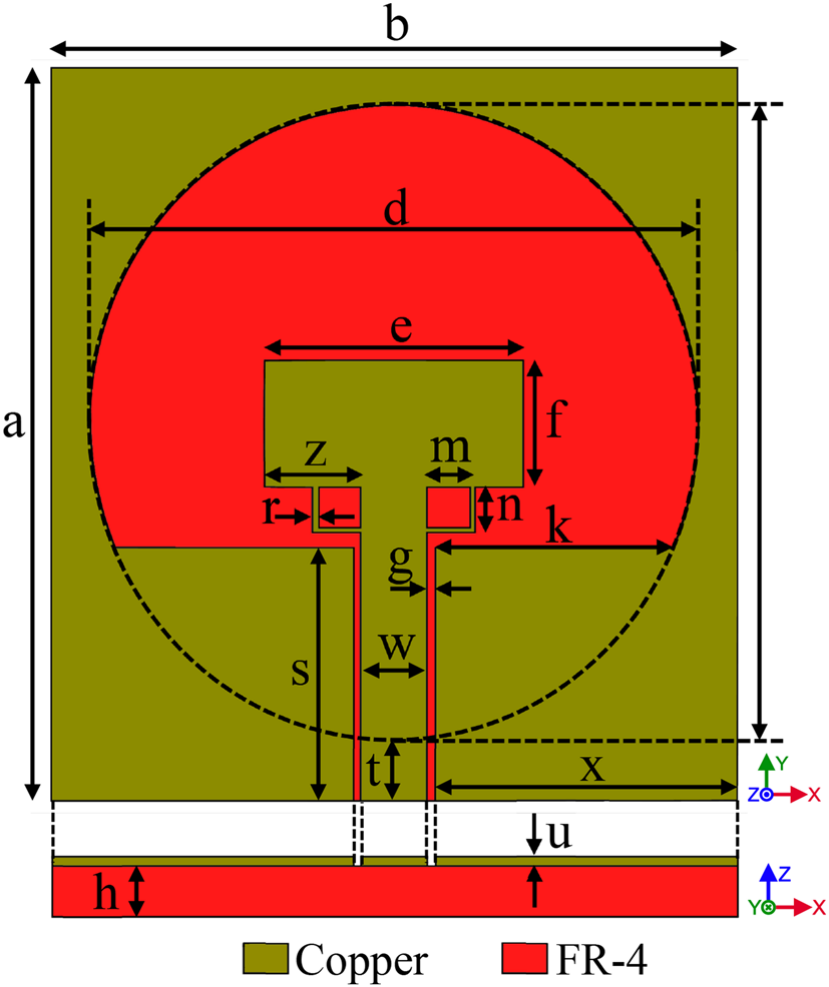
The final antenna design on the FR4 substrate.

As a method of achieving this goal based on prior research (Fig. 1C), we attempted to extend the ground plane of the antenna. As shown in Fig. 1D an internal circle cut off antennas ground plane for increasing the bandwidth impedance was done. This changes the volume of the capacitor between the radiation patch and the ground plane, As a result, the antenna's operating frequency, as illustrated in Fig. 2d, increases from 2.8 to 7.1 GHz. The next section creates a connection between the radiation patch and feeding-line in order to increase the antennas' bandwidth using two symmetric rings with a width of 0.2 mm. As seen in Fig. 1E, two rings with the forms of symmetric rectangles are implanted in both sides of the feeding-line and bottom patch of the antenna in order to increase antenna's bandwidth. With such a structure, the antenna's bandwidth can be magnificently expanded by precisely adjusting the length, width, and thickness of these two rings. The antenna's bandwidth can reach 22 GHz by using these two rings' optimal dimensions of length, width, and thickness (from 3 to 25 GHz). The antenna's final design is shown in Fig. 3, and its dimensions are illustrated in Table 1.

**Table 1:** Final antenna design on FR4 substrate.

Parameters	Dimension (mm)	Parameter	Dimension (mm)
a	29	m	1.7
b	27	n	1.8
c	25	r	0.2
d	24	s	1
e	10.2	t	2.5
f	5	u	0.01
g	0.3	w	2.6
h	1.6	x	11.9
k	9.4	z	3.8

**Figure 4:**
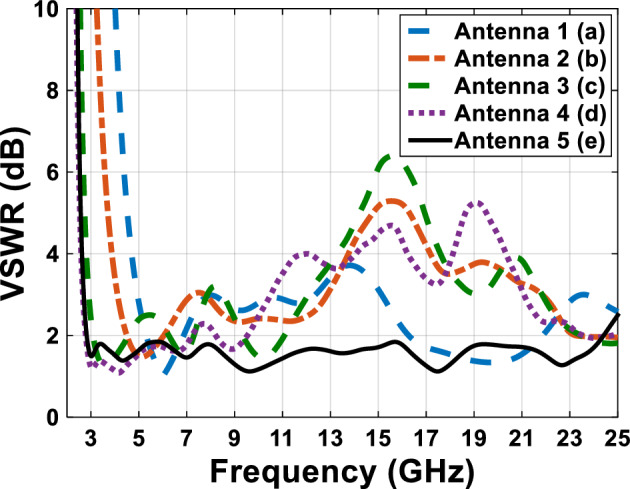
The comparing VSWR graphs of Fig. 1 antennas.

**Figure 5:**
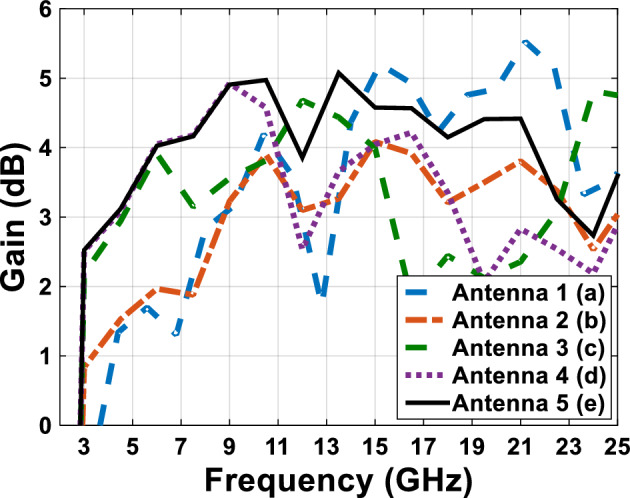
The comparing gain graphs of Fig. 1 antennas.

**Figure 6:**
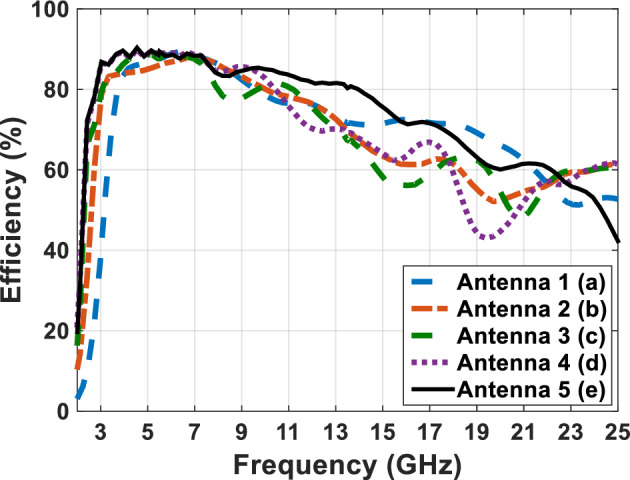
The comparing efficiency graphs of Fig. 1 antennas in present.

Figure 4 shows a comparative plot of the voltage standing wave ratio (VSWR) of the designed antennas in Fig. 1. According to VSWR curves, all antennas working frequencies are under 2 dB, this shows the compatibility of the proper impedance of the designed antenna. Figures 5 and 6 show the comparative plot of antenna’s gain and total interest of antennas designed in Fig. 1. According to the comparison chart in Fig. 5, the gain value of the antenna in Fig. 1 is between 2 and 5.5 dB, which is a desirable value.

In figure Fig. 6, we can see the comparative diagram of the efficiency antenna. Antenna efficiency is the amount of power that reaches the desired antenna divided by the amount of antenna power radiated. A high-efficiency antenna radiates a large amount of input power to the antenna, but a low-efficiency antenna absorbs a large amount of power as internal losses or reflected due to impedance mismatch. In Fig. 6, the average efficiency of the antenna is more than 50%, which shows that the antenna radiates the input power well throughout the frequency band and the input power has little loss, which shows that the designed antenna has a good impedance matching.

### Designing antenna on FR4 substrate

**Figure 7:**
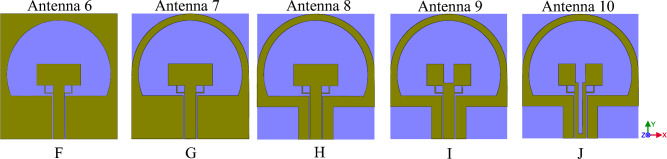
The designing processes of SWB antenna respectively form (**F**) to (**J**) are depicted.

**Figure 8:**
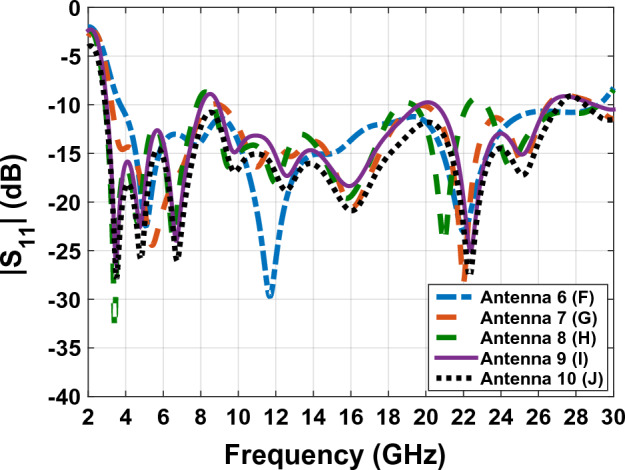
The comparing scattering parameters graphs of Fig. 7 antennas.

In this section, we use a substrate from FR4 to plexi-glass the antenna will be transparent and allow the light to pass through it. Plexi-glass has high resistance against different climates. It is cheap, and therefore a good choice to be used as a lucid substrate. We used the usual plexi-glasses available in the market, with $\mu =1$, ${\varepsilon }_{r}=2.7$ and 1 mm thickness has been used. In Fig. 7, the designing processes of the SWB antenna respectively, from F to J, are depicted. According to the designed antenna in "Designing antenna on FR4 substrate", in this section, we try to achieve more transparency by changing the substrate to plexi-glass. The FR4 substrates are made of Glass Wool in different resins, this layer has many advantages such as thermal, electric and mechanical properties. Moreover, it has a better RF performance and, it can be used in frequencies from 1 to 10 GHz. When this layer is used in frequencies above 10 GHz, it has a negative effect and causes wave interference and mismatch problems. As such, it is a good choice to use plexi-glass instead of FR4 as a substrate. In Fig. 7F in comparison to Fig. 3 we have only compared the proxy glass to FR4. According to the graph parameters in Fig. 8, we can see that the Bandwidth has not changed noticeably. In further steps, we have eliminated the metal part in order to reach more transparency to gain a higher light pass.

**Figure 9:**
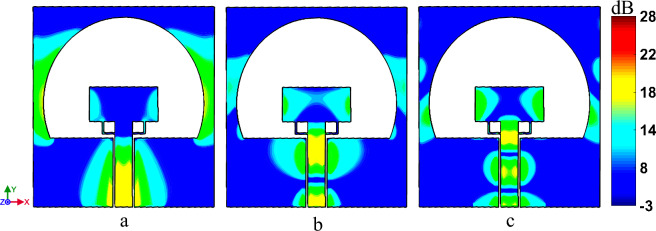
The antenna surface currents of Fig. 7F at (**a**) = 6 GHz, (**b**)  = 14 GHz and (**c**) = 22 GHz frequencies.

In Fig. 9, the surface currents at three operating frequencies, 6, 14, and 22 GHz, have been investigated. According to Fig. 9, which shows the electrical current of the patch and antenna's ground plane, we can see that removing parts with low electrical surfaces current has little effect on electromagnetic wave radiation. In addition, the bandwidth increases magnificently with a ratio of 1:10, making the antennas applicable for SWB applications. As a result, according to Fig. 8, the final working frequency range of Fig. 7J antenna has reached from 2.9 to 27.2 GHz.

**Figure 10:**
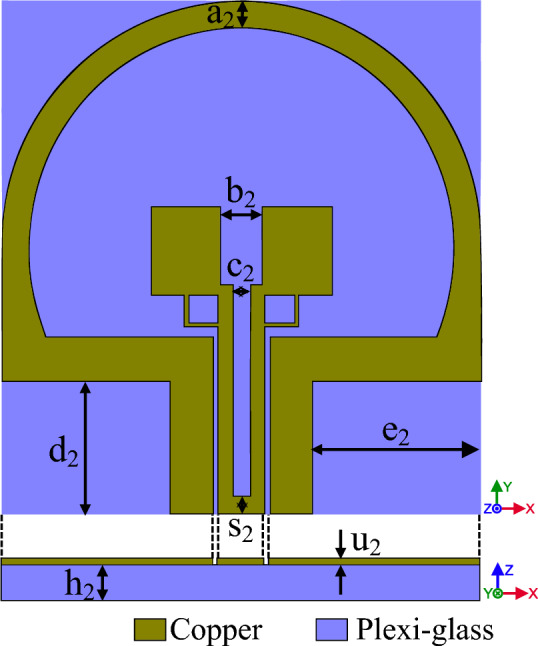
The final designed antenna on plexi-glass substrate.

**Figure 11:**
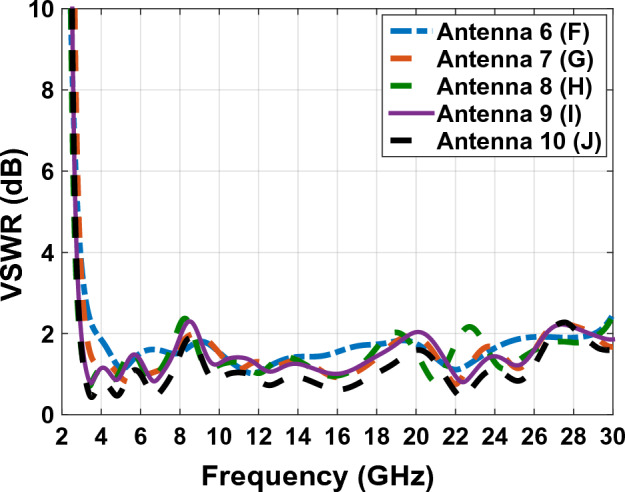
The comparing VSWR graphs of Fig. 7 antennas.

In Fig. 10 the final product of the antenna with a transparent substrate is shown and Table 2 represents the dimension of the antenna. Figure 11 shows, the comparative chart of VSWR of Fig. 7. According to the VSWR all the antenna’s frequencies are under 2 dB which, shows suitable impedance adaptability. Also, in Fig. 11, the diagram of Fig. 7G is below 2 dB throughout the operating frequency from 2.9 to 27.2 GHz, indicating the performance and good impedance matching of the antenna.

**Table 2:** The dimension’s parameters of the antenna in Fig. 10.

Parameter	Dimension (mm)	Parameters	Dimension (mm)
a_2_	1.5	e_2_	9.5
b_2_	2.4	s_2_	1
c_2_	1	h_2_	1
d_2_	7.5	u_2_	0.04

### Adding a solar panel to the antenna with the transparent substrate

**Figure 12:**
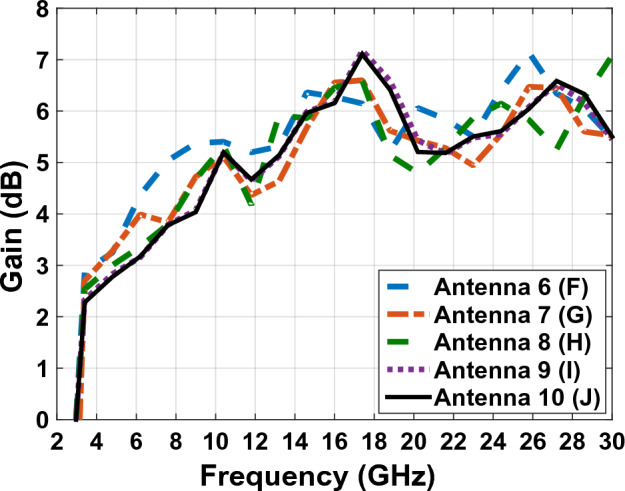
The comparing gain graphs of Fig. 1 antennas.

**Figure 13:**
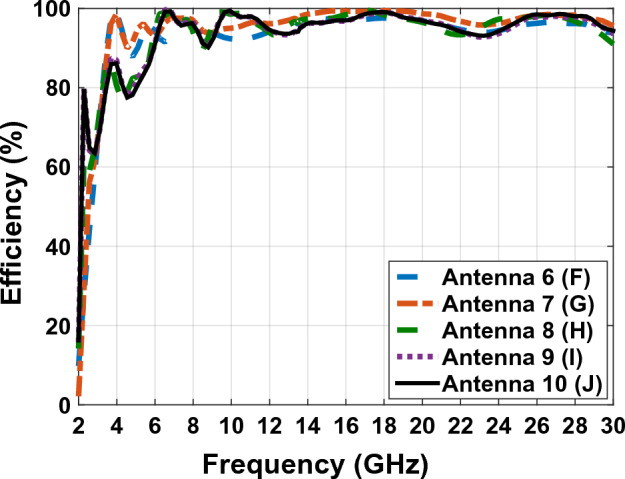
The comparing efficiency graphs of Fig. 1 antennas in present.

Figures 12 and 13 show the comparative chart of antenna gain and the comparative chart of antenna efficiency of Fig. 7 antennas, respectively.

According to the diagram in Fig. 12, the peak gain value of the final antenna of Fig. 7J is 7.2 dB at a frequency of 17.5. The comparative diagram of Fig. 12 shows that the changes in the antennas of Fig. 7 do not have negative effects on the antenna gain.

**Figure 14:**
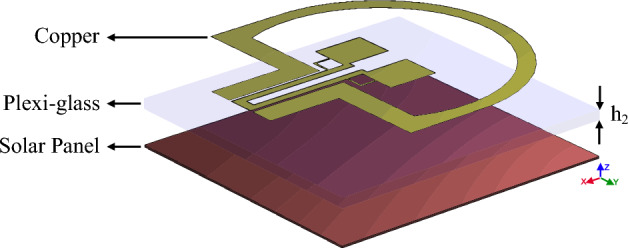
Schematic of placing the solar panel under the Antenna Fig. 10.

The efficiency of the antennas in Fig. 7 is analyzed in Fig. 13, which shows a value above 90% throughout the frequency band. By comparing the efficiency diagram of Fig. 13 (antenna with plexi-glass substrate) and the efficiency diagram of Fig. 6 (antenna with FR4 substrate), we can conclude that the FR4 substrate has a decreasing efficiency at a frequency above 10 GHz, and this confirms the poor performance of the FR4 substrate at frequencies above 10 GHz. But, in the antenna with the plexi-glass substrates, efficiency is higher than 90% over the antenna's operative frequency band, which justifies the usage of the plexi-glass layer rather than the FR4 substrates. In the previous phase, according to the defined application of the antenna and the optimal use of solar layers on the antenna shown in Fig. 10 effected on the antenna in Fig. 11, the radiation metal parts of the antenna with less surface current are cut and reduced. More light will be able to pass through the transparent plexi-glass substrate as a result, and the solar cell placed under the substrate will also be exposed to sunlight. According to the calculations, the surface of the antenna is 783 mm, and also the whole surface of the metal layer is approximately 69.2 mm which is around 36.7 percent of the whole plexi-glass laid on the copper sheet. Therefore 63.3 percent of the sun’s rays pass through the plexi-glass and reach to the solar panel. As shown in Fig. 14, the solar panel with the electrical permeability constant ${\varepsilon }_{r}=1.5$ and $\mu =1$ with a height of 0.2 mm is used under the plexi-glass^29,30^.

**Figure 15:**
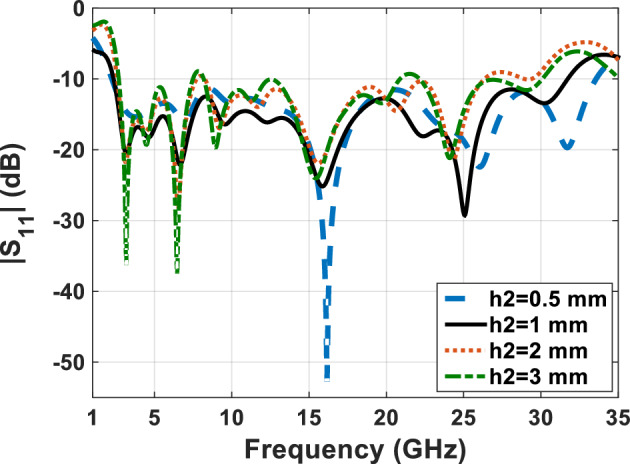
Comparison chart of scattering parameters for different thicknesses of substrate.

**Figure 16:**
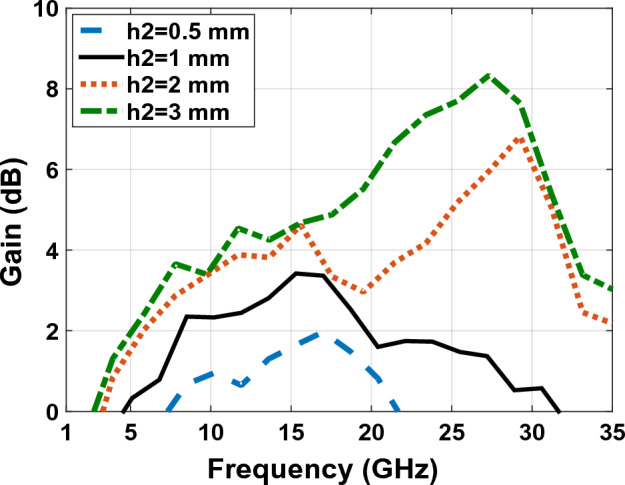
Comparison chart of gain for different thicknesses of substrate.

**Figure 17:**
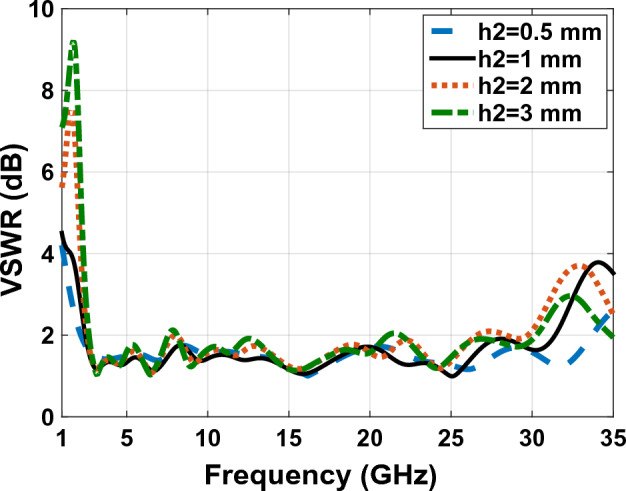
Comparison chart of VSWR for different thicknesses of substrate.

**Figure 18:**
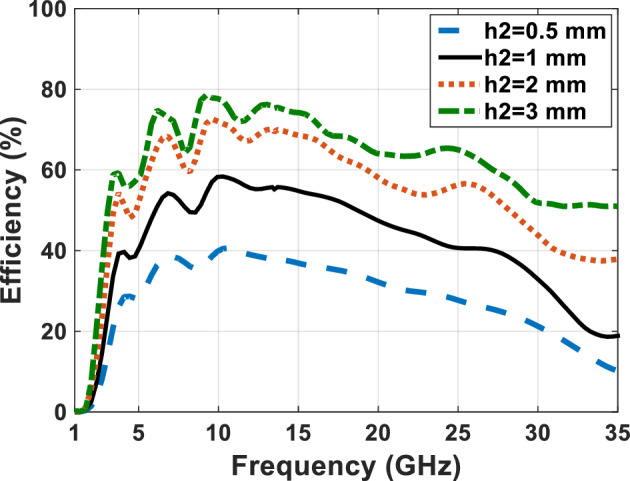
Comparison chart of efficiency for different thicknesses of substrate.

With respect to the availability of plexi-glass in different sizes in the market, we have used different sizes of plexi-glass (${h}_{2}$) as shown in comparative charts in Figs. 15, 16, 17 and 18, respectively. These comparative charts of scattering factor, gain, VSWR and efficiency along with solar cells for the different plexi-glass thickness ${h}_{2}$ (3 mm, 2 mm, 1 mm, 0.5 mm) are shown.

With respect to the availability of plexi-glass in different sizes in the market, we have been able to use different sizes of plexi-glass (${h}_{2}$) as shown in comparative charts in Figs. 15, 16, 17 and 18, respectively. These comparative charts of scattering factor, gain, VSWR and efficiency along with solar cells for the different plexi-glass thickness ${h}_{2}$ (3 mm, 2 mm, 1 mm, 0.5 mm) are shown.

According to the comparative diagram ${\mathrm{S}}_{11}$ in Fig. 15, it can be concluded that placing the solar cell under the antenna has increased the bandwidth of the antenna. This shows that the solar cell can exert positive effects on increasing the bandwidth of the antenna. This bandwidth was obtained at its best when the substrate is 0.5 mm thick. This bandwidth is from 2.7 to 34.2 GHz and has been measured. It can also be said that in addition to the positive changes of the solar cell on the bandwidth of the antenna, the reduction of the substrate thickness can also have a positive effect on improving the bandwidth of the antenna.

Figure 16 shows the comparative diagram of the antenna gain after placing the solar cell under the antenna. The diagram in Fig. 16 is measured according to the thickness of different substrates (0.5 mm, 1 mm, 2 mm, 3 mm). By examining the gain diagram in Fig. 16, it can be concluded that placing the solar cell as a whole can cause a slight decrease in gain, and on the other hand, increasing the thickness of the substrate can significantly increase the gain. The best gain of the antenna occurs when the thickness of the substrate is 3 mm, and the peak gain value in this case is 8.1 dB. Figure 17 shows, the comparative chart of VSWR of Fig. 14. According to the VSWR all the antenna Fig. 7 frequencies are under 2 dB which, shows the suitable impedance adaptability.

Figure 18 shows the comparative diagram of antenna efficiency after placing the solar cell under the antenna. The diagram in Fig. 18 is measured according to the thickness of different substrates (0.5 mm, 1 mm, 2 mm, 3 mm). By examining the efficiency diagram in Fig. 18, it can be concluded that placing the solar cell as a whole can cause a slight decrease in efficiency, and in contrast to increasing the thickness of the substrate, it can significantly increase the efficiency. The best efficiency of the antenna occurs when the thickness of the substrate is 3 mm and the peak efficiency in this case, is 80%.

The scattering parameter graph in Figs. 8 and 15, the gain graph in Figs. 12 and 16, and the efficiency graph in Figs. 13 and 18 all allow us to draw the conclusion that the solar panel has a small negative effect on the antenna's gain and a large positive effect on the antenna's bandwidth.

## Time domain characteristics

Time transit response characteristics of wideband antenna is in accordance with time domain characteristics which has special importance. In fact, in addition to the sufficient bandwidth impedance, non distorted and un-scattered pulse attitude is required for wave transfer and quality and less dispersion are crucial for wave transfer.

For the plus loss parameters, the correlation coefficient is defined^31^. The correlation coefficient, the maximum correlation between two signals is from time delay τ, so this parameter shows the similarity between the transmitted pulse and the received pulse. The maximum correlation coefficient is equal to one, which shows the complete similarity of received and input signals.

The time correlation coefficient is calculated from Eq. (4):

4 $$Fidelity=max\frac{\underset{-\infty }{\overset{+\infty }{\int }}{s}_{t}\left(t\right){s}_{t}\left(t-\uptau \right)\mathrm{dt}}{\sqrt{\underset{-\infty }{\overset{+\infty }{\int }}{\left|{s}_{t}(t)\right|}^{2}dt \underset{-\infty }{\overset{+\infty }{\int }}{\left|{s}_{r}(t)\right|}^{2}dt}}$$

which ${S}_{t}$ is transmitter and ${S}_{r}$ as receiver in Table 3 the similar work has been shown.

**Table 3:** Correlation coefficients of different references.

References	^32^	^33^	^34^	^35^	^36^
Correlation coefficient	0.8	0.92	0.939	0.8415	0.8754

Normalization of ${T}_{X}$ and ${R}_{X}$ pulses are performed using Eqs. (5) and (6)^37^. Where ${T}_{s}^{n}$ is recognized as the normalized ${T}_{X}$ pulse, and ${R}_{s}^{n}$ is recognized as the normalized ${R}_{X}$ pulse. The amplitude of the ${R}_{X}$ pulse is less than the ${T}_{X}$ pulse, so the normalization has been performed only to find similarity between the wave shapes of the pulses. The fidelity factor equation is calculated using point to point cross correlation between the normalized pulses as follows: Eq. (7)^37^:

5 $${T}_{s}^{n}=\frac{{T}_{s}(t)}{\sqrt{ \underset{-\infty }{\overset{\infty }{\int }}{\left|{T}_{s}(t)\right|}^{2}dt}}$$

6 $${R}_{s}^{n}=\frac{{R}_{s}(t)}{\sqrt{ \underset{-\infty }{\overset{\infty }{\int }}{\left|{R}_{s}(t)\right|}^{2}dt}}$$

7 $$FF=max\underset{-\infty }{\overset{\infty }{\int }}{T}_{s}^{n}\left(t\right) {R}_{s}^{n}\left(t\right) \left(t+\tau \right) dt$$

**Figure 19:**
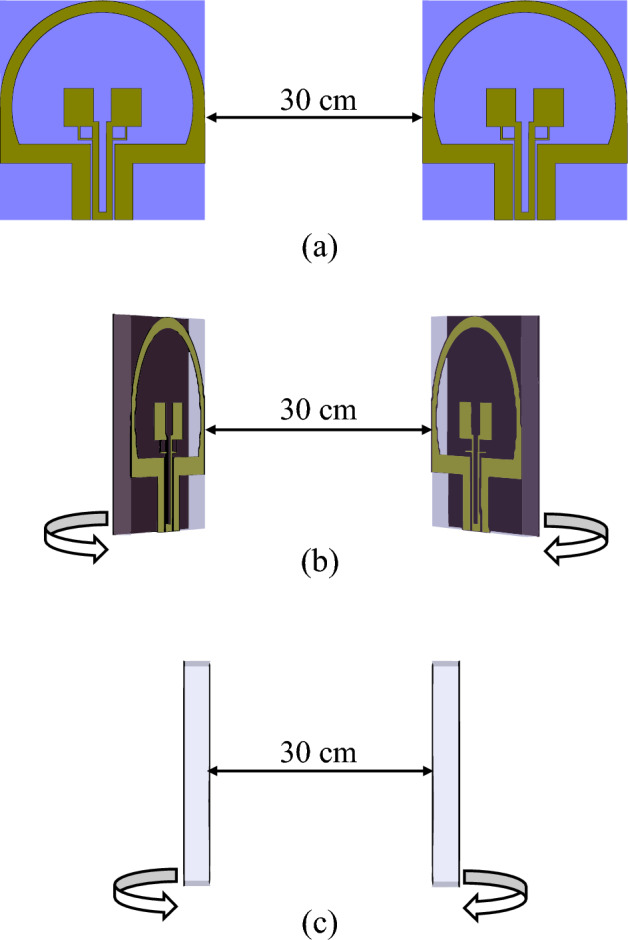
Two similar antennas across each other with 30 cm distance. In parts (**a**–**c)**, φ takes values 0°, 45° and 90°, respectively ($a={\mathrm{\varphi }0}^{^\circ }$, $b={\mathrm{\varphi }45}^{^\circ }$, $a={\mathrm{\varphi }90}^{^\circ }$).

**Figure 20:**
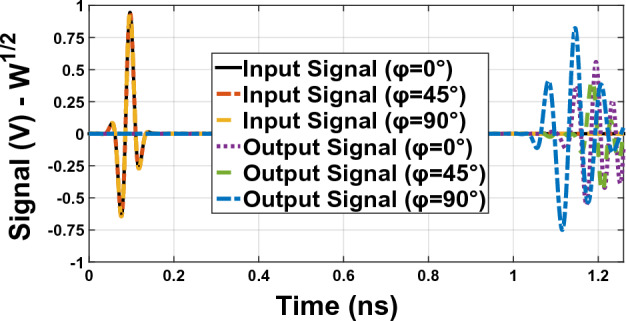
Comparison diagram of input and output correlation coefficient in Fig. 19 in $\mathrm{\varphi }={0}^{^\circ }$,$\mathrm{\varphi }={45}^{^\circ }$ and $\mathrm{\varphi }={90}^{^\circ }$.

For calculating the correlation coefficient, we choose the wide band Gaussian pulse as the input transmitter signal for the antenna, As shown in Fig. 19. Transmitter antenna and receiving antenna are 30 cm apart at different angles φ [${0}^{^\circ }$, ${45}^{^\circ }$, ${90}^{^\circ }$] degrees in XZ panel. In Fig. 20, you can see the graphs of the correlation coefficient of the antenna with the solar panel.

In addition to correlation coefficient, the calculation of the antenna group delay which is gained by measuring parameter delay ${S}_{21}$ is also measured. This is one of basic requirements of wide band antennas. The correlation coefficient of un scattered behavior with keeping linear phase which is proper to constant group delay is gained^35^. In other words, if in practice the group delay is approximately constant it provides the characteristics for the acceptable PULS, Eqs. (8) and (9)^26^.

8 $${\tau }_{g}\left(\omega \right)=-\frac{d\theta \left(\omega \right)}{d\omega }={t}_{TX}+{t}_{D}+{t}_{RX}$$

9 $${t}_{D}=\frac{D}{c}$$

In Eq. (8), $\omega$ is the angular frequency, $\theta (\omega )$ is the frequency in the observed point and in Eq. (9) c is the light velocity, ${t}_{D}$ is the wave emission transmission time between the two antennas which are a D distance apart. ${t}_{TX}$ and ${t}_{RX}$ are the time delay of the transmitter and receiver. For the two similar antennas, ${t}_{TX}$ and ${t}_{RX}$ are equal, if the phase shift is proper to the frequency the group delay is constant and therefore there is no dispersion, otherwise the signal would have distortion. Increasing the zero point and sudden mutations in the frequency range has a negative effect on dispersion characteristics^38^. Group delay waves are related to weal Bandwidth application.

**Figure 21:**
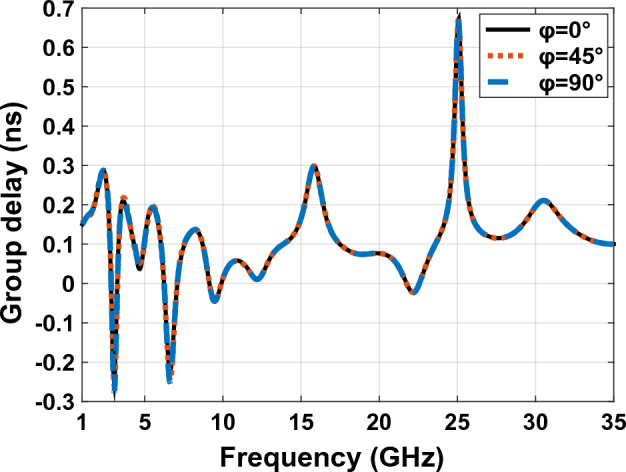
The group delay graph for two antennas across each other in Fig. 19.

Two similar antennas across each other with 30 cm distance. in parts a, b and c, takes values 0, 45 and 90 degrees respectively according to Fig. 19. The result of group delay calculation by CST MWS software is shown in Fig. 21. With respect to the group in Fig. 21, the answer to group.delay is suitable value, because the group delay should be $-1ns\le {D}_{t}\le +1ns$ which is the minimum amount, the maximum standard amount for the group delay is obtained from equations number 10, acceptable amount is equal 3.8 ns^39^.

10 $${t}_{D}=\frac{{f}_{s}}{2}$$

**Figure 22:**
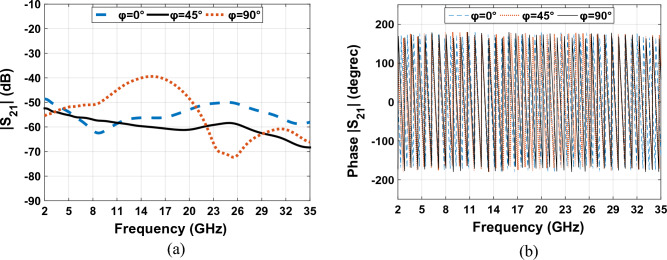
The scattering parameters and phase responses graphs for Fig. 19 in $\mathrm{\varphi }={0}^{^\circ }$,$\mathrm{\varphi }={45}^{^\circ }$ and $\mathrm{\varphi }={90}^{^\circ }$. (**a**) isolation achieved for the design. (**b**) Phase response ${S}_{21}$ of the proposed design.

The graph showing the phase variation of Fig. 19 in Fig. 22, illustrates that the given phase ${S}_{21}$ has a constant variation and is linear and therefore it has less distortion loss by orientation. This means that by changing the direction of the transmitter and receiver antenna, we transfer a good coupling from the transmitter antenna to the receiver antenna, and as a result, we have perfect wave transmission at various angles.

## MIMO antenna performance analysis

**Figure 23:**
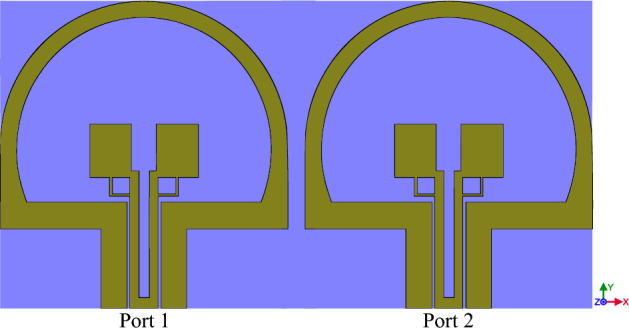
The MIMO design of antenna Fig. 14.

Multiple-input-multiple-output (MIMO) is a method of wireless communication systems using multiple transmission and receiving antennas. With this feature, the user can use two or more antennas to reduce the sending and receiving information delay and increase the speed of communication using the specified channel. In this section as it is shown in Fig. 23, in order to analyze the performance of the MIMO antenna, we have placed two designed antennas in Fig. 14 together, then to confirm the proper performance the of designed antenna The basic parameters of MIMO antenna such as envelope correlation coefficient (ECC), diversity gain (DG), mean effective gain (MEG), total active reflection coefficient (TARC) and channel capacity loss (CCL) are investigated.

### Envelope correlation coefficient (ECC)

**Figure 24:**
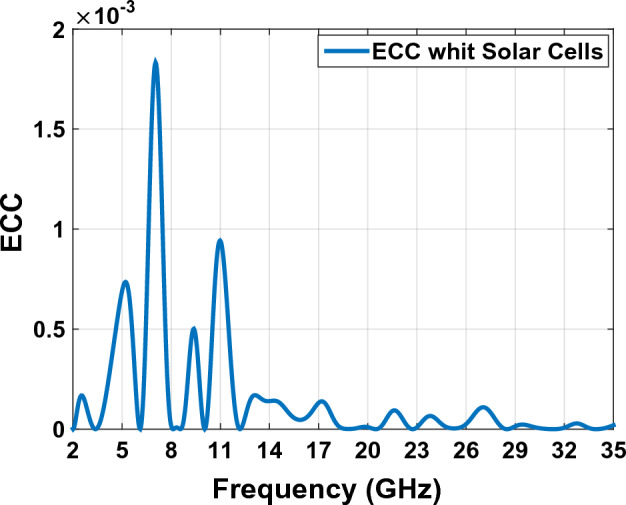
The ECC graph of MIMO antenna Fig. 23.

EEC is an important point of strength for every MIMO antenna^40^. This measure can be computed using complex patterns (Eqs. 11, 12). These parameters considers radiation patterns of the antenna when two or more antenna are operating simultaneously. Figure 24 shows the ECC plot of the designed antenna in Fig. 23. In an ideal situation EEC improves as it approaches to zero. The ECC value is calculated between the numbers 1 and 0, ECC = 0 means the antennas are completely decoupled, while ECC = 1 means they are essentially short-circuited. A correlation below about 0.3–0.4 is usually considered "good enough" for MIMO. The maximum value of ECC in the diagram of Fig. 24 is 0.002, which is a very good value. Increment in channel capacity and diversity gain are the results of this improvement^41^.

11 $$\rho _{e} = \frac{{\left| {\iint_{{4\pi }} {\left[ {\overset{\lower0.5em\hbox{$\smash{\scriptscriptstyle\rightharpoonup}$}} {E} _{1} \left( {\theta \cdot \emptyset } \right) \cdot \overset{\lower0.5em\hbox{$\smash{\scriptscriptstyle\rightharpoonup}$}} {E} _{2} \left( {\theta \cdot \emptyset } \right)} \right]d\Omega }} \right|^{2} }}{{\iint_{{4\pi }} {\left| {\overset{\lower0.5em\hbox{$\smash{\scriptscriptstyle\rightharpoonup}$}} {E} _{1} \left( {\theta \cdot \emptyset } \right)} \right|^{2} d\Omega }\iint_{{4~\pi }} {\left| {\overset{\lower0.5em\hbox{$\smash{\scriptscriptstyle\rightharpoonup}$}} {E} _{1} \left( {\theta \cdot \emptyset } \right)} \right|}^{2} d\Omega }}$$

12 $$\overset{\lower0.5em\hbox{$\smash{\scriptscriptstyle\rightharpoonup}$}} {E} _{1} \left( {\theta \cdot \emptyset } \right) \cdot \overset{\lower0.5em\hbox{$\smash{\scriptscriptstyle\rightharpoonup}$}} {E} _{2} \left( {\theta \cdot \emptyset } \right) = \overset{\lower0.5em\hbox{$\smash{\scriptscriptstyle\rightharpoonup}$}} {E} _{{\theta 1}} \left( {\theta \cdot \emptyset } \right) \cdot \overset{\lower0.5em\hbox{$\smash{\scriptscriptstyle\rightharpoonup}$}} {E} _{{\theta 2}}^{*} \left( {\theta \cdot \emptyset } \right) + \overset{\lower0.5em\hbox{$\smash{\scriptscriptstyle\rightharpoonup}$}} {E} _{{\emptyset 1}} \left( {\theta \cdot \emptyset } \right) \cdot \overset{\lower0.5em\hbox{$\smash{\scriptscriptstyle\rightharpoonup}$}} {E} _{{\emptyset 2}}^{*} \left( {\theta \cdot \emptyset } \right)$$

### Diversity gain (DG)

**Figure 25:**
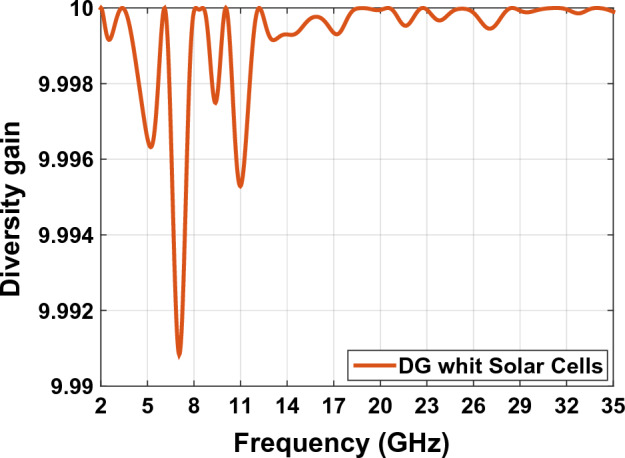
The DG graph of MIMO antenna Fig. 23.

Increasing diversity gain is a measure of the impact of diversity on communication systems^42^. When diversity occurs, a transmitter receives multiple signals from channels with different sources in MIMO systems. For better signal reception diversity gain should be more than EEC. In MIMO systems the more antenna we use, the more combines power in system receives diversity. The more diversity is the lower correlation coefficient will be. Diversity gain can be calculated using Eqs. (13) and (14)^43^. Figure 25 shows DG curve of the antenna designed in Fig. 23:

13 $$\mathrm{DG}=10{e}_{p}$$

14 $${\mathrm{e}}_{p}=\sqrt{1-{\left|\rho \right|}^{2}}$$

### Mean effective gain (MEG)

**Figure 26:**
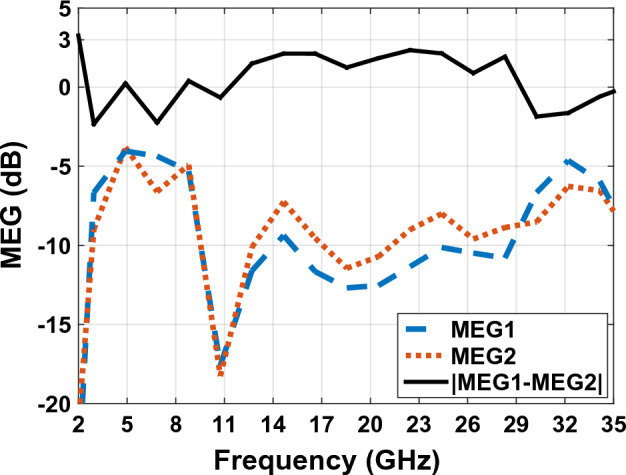
The ${MEG}_{1}$, ${MEG}_{2}$ and $\left|{\mathrm{MEG}}_{1}-{\mathrm{MEG}}_{2}\right|$ graph of MIMO antenna Fig. 23.

Mean effective gain MEG is a criterion which is defined in a wireless environment of the antenna^44^. This technique is also discussed in^45^. MEG in^46^ is calculated from the results of the S parameter using Eq. (15), where M is the total number of antennas^47^. Figure 26 shows the measured MEG of the designed antenna in Fig. 23. Also, the power ratio (k), which is the difference in MEG magnitude, is calculated using Eq. (16). Here the value of k (power ratio) is very close to 0 dB. When this value is less than 3 decibels, it means that there is no significant difference between the average received power in the MIMO antenna^48^.

15 $${MEG}_{i}=0.5 {\eta }_{i.rad}=0.5\left[\sum_{j=1}^{M}{\left|{S}_{ij}\right|}^{2}\right]$$

16 $$k=\left|{MEG}_{1}-{MEG}_{2}\right|<3 dB$$

### Total active reflection coefficient (TARC)

**Figure 27:**
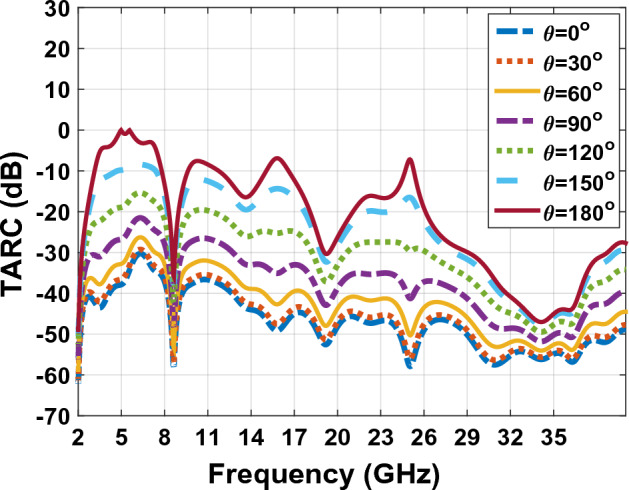
The TRAC graph of MIMO antenna Fig. 23 with $\theta$ from ${0}^{^\circ }$ to ${180}^{^\circ }$.

TARC is defined as the square root ratio of the total reflective Power divided by the root of the total power in a multi-port antenna system^49^. TARC is a method to adjust parameters variation for all MIMO ports. This method also constructs a curve that represents the effect of the power supply phase on antenna gates^42^. It is desirable to minimize the TARC value to avoid excessive energy losses due to reflections (less than − 10 dB). Lower TARC values indicate better antenna performance and improved efficiency. TARC values is verified using a single phase and randomly, on the interval [0–180] degree^46^. Figure 27 represents the measured TARC of the antenna designed in Fig. 23. Results from Fig. 27 shows that TARC covers the antenna proposed in Fig. 23. For designed MIMO antenna with two ports TARC can be achieved using relation (17)^46^.

17 $$\tau =\frac{\sqrt{(({\left|{S}_{11}+{S}_{12}{e}^{j\theta }\right|}^{2}+\left({\left|{S}_{21}+{S}_{22}{e}^{j\theta }\right|}^{2}\right))}}{\sqrt{2}}$$

### Channel capacity loss (CLL)

**Figure 28:**
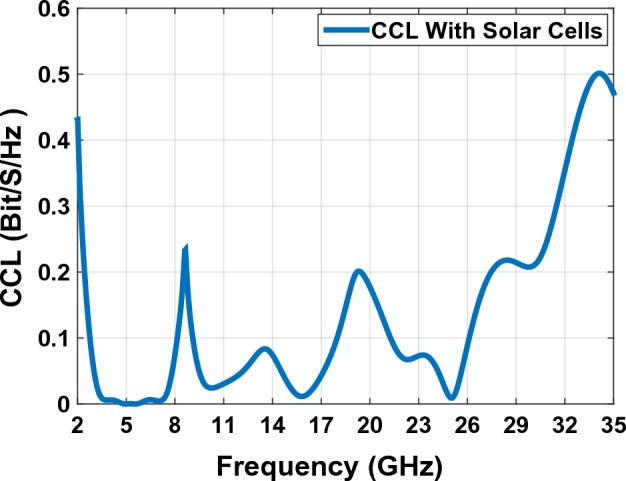
The ECC graph with Solar Cells of MIMO antenna Fig. 23.

MIMO antennas, for using huge amount of data in a limited channel and supplied channel capacity is the maximum data rate through which through which the signal can pass using MIMO channel. CCL is an important criterion for analyzing and verifying channel capacity functionality. The CCL parameter provides details of the wasting capacity of the systems channel along the correlation effect. CCL is calculated numerically using Eqs. (18), (19), (20) and (21). The MIMO antennas CCL should be less than 0.4 bpsHz in antenna’s operating frequency. Figure 28 shows that for the proposed MIMO antenna in Fig. 23, the obtained CCL in all the operating frequencies is less than 0.25 Bit/S/Hz^50,51^. This value is less than the standard practical 0.4 Bit/S/Hz for the antenna working frequency and this fact confirms the high throughput of the proposed antenna.

18 $$C\left(loss\right)={-log}_{2}\mathrm{det}\left(a\right)$$

where a is the correlation matrix:

19 $$a=\left[\begin{array}{cc}{\sigma }_{11}& {\sigma }_{12}\\ {\sigma }_{22}& {\sigma }_{21}\end{array}\right]$$

20 $${\sigma }_{ii}=1-\left({\left|{S}_{ii}\right|}^{2}-{\left|{S}_{ij}\right|}^{2}\right)$$

21 $${\sigma }_{ij}=1-\left({{S}_{ii}}^{*}{S}_{ij}+{{S}_{ji}{S}_{jj}}^{*}\right)$$

## Analysis the measurement of the manufactured antenna

**Figure 29:**
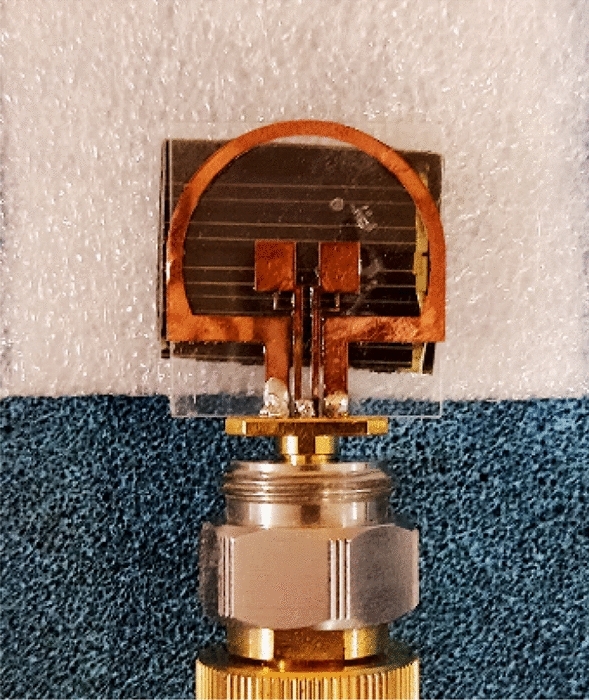
Antenna manufactured with a transparent substrate and placing a solar cell along with a $50\Omega$ coaxial SAM port.

In previous researches, metal oxide has been used to make the radiating part of the antenna. Due to its low conductivity, this oxide creates interference in the operation process and radiation pattern of the antenna. In addition, the difficulty of production and costly manufacturing have been among the problems of this method. We have used copper sheet for the antenna’s ground and radiating sections due to its availability, low cost, and convenience of fabrication, as well as the fact that lines with low resistance attenuate signals less than lines with high resistance. These sheets are cut by CNC laser machine with 0.02 mm precision. Since copper sheets come in a variety of thicknesses, the simulation shows that the with a thickness of 0.04 mm for a sheet, is the most suitable thickness to take into account for antenna building. Also, according to the simulation results, the thickness of Plexi-glass is considered to be 1 mm. Figure 29 shows a sample of a transparent antenna with a solar panel and a solar panel bead that has been manufactured. This antenna is made with a coaxial SubMiniature version A (SMA) connector with plexi-glass layer and CPW feeding line.

**Figure 30:**
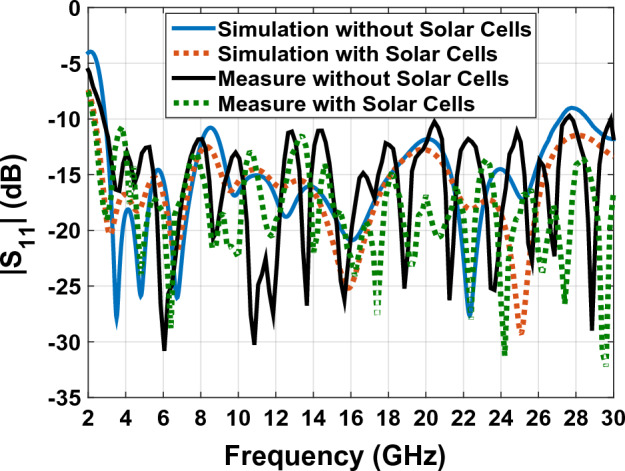
The comparison diagram of the dispersion parameter ($${S}_{11})$$ of the simulated antenna with the built sample.

**Figure 31:**
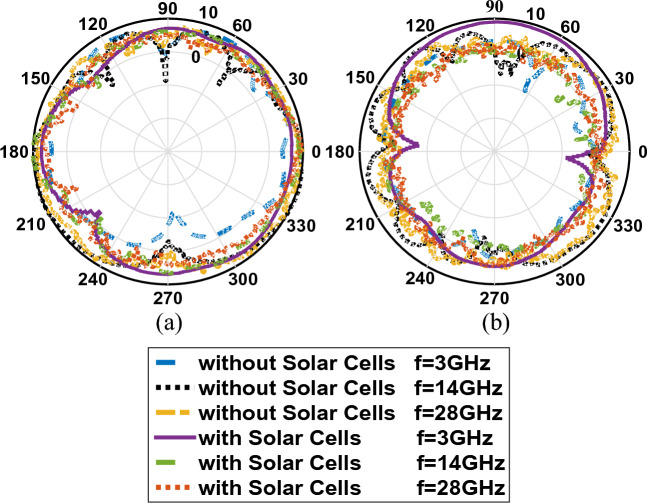
The radiation patterns of manufactured antenna. (**a**) Co-pol, (**b**) Cross-pol.

Figure 30 shows the comparison diagram of the dispersion parameter of the simulated antenna with the built sample. According to this comparison, the bandwidth of the antenna in the construction and simulation shows the values between 2.9 and 29.2 GHz, which provides us with the bandwidth of SWB programs. In Fig. 31, according to the antenna pattern made in both Co and Cross modes, we can conclude that we will have an omnidirectional pattern.

The proposed antenna is compared with previously reported ultra wideband (SWB) antennas in terms of electrical dimensions, operating frequency range, operating bandwidth (BW), bandwidth aspect ratio (BDR) peak gain and substrate material. Table 4 presents the results. The aspect ratio of the bandwidth, which is represented by the percentage unit and determined by Eq. (22), reveals how much the operational bandwidth is in the unit of the antenna’s electric surface^6^.

22 $$BDR=\frac{BW\%}{{L}_{{f}_{low}}\times {W}_{{f}_{low}}}=\frac{BW\%}{{\lambda }_{l}*{\lambda }_{w}}$$

where ${L}_{{f}_{low}}$ indicates the electrical length, ${W}_{{f}_{low}}$ It shows the electrical dimensions (width) of the antenna in the working band frequency. where ${\lambda }_{l}$ and ${\lambda }_{w}$ are the wavelengths associated with the lower cut of frequency ${f}_{l}$, and it is calculated using the following Eq. (23):

**Table 4:** A comparison between the basic characteristics of the proposed antenna such as electrical dimensions, operating frequency range, operating bandwidth (BW), bandwidth aspect ratio (BDR), peak gain and the following material with previously reported ultra-wideband antennas.

References	Size (mm^2^)	Freq. range (GHz)	BW (%)	BDR	Peak gain (dB)	Substrate material
^52^	0.16 λ × 0.12 λ	0.4–20	192	10,000	6.3 at 17 GHz	FR-4
^53^	0.4 λ × 0.25 λ	3–20	147	1470.0	6 at 6 GHz	FR-4
^54^	1.28 λ × 4.9 λ	24–40	68	3429.26	19 at 34 GHz	Rogers RT5880
^55^	0.24 λ × 0.32 λ	2.4–28.4	169	2200.5	7 at 18 GHz	FR-4
^56^	0.30 λ × 0.285 λ	2.25– 11.05	132	1547.7	5.05 at 10 GHz	FR-4
^57^	0.32 λ × 0.32 λ	0.64–16	184	1802.7	10 at 9 GHz	FR-4
^58^	0.33 λ × 0.416 λ	2.5–110	191	1391.0	6 at 40 GHz	FR-4
^59^	0.18 λ × 0.22 λ	1.30–20	175	4261.0	5 at 20 GHz	Rogers RT5880
^60^	0.17 λ × 0.13 λ	0.96–10.9	167	6975.2	4.5 at 10 GHz	Rogers RT5880
^61^	0.38 λ × 0.55 λ	3–35	168	503.8	6 at 13 GHz	FR-4
^62^	0.326 λ × 0.265 λ	3.06–35	168	1944.6	Not reported	${\varepsilon }_{r}=2.22$ $tan\delta =0.0009$
^63^	0.32 λ × 0.34 λ	3.4–37.4	166	1531.8	11 at 32.5 GHz	Rogers RT5880
^64^	0.31 λ × 0.46 λ	3.15–32	164	1102.9	3.2 at 5 GHz	FR-4
^65^	0.278 λ × 0.234 λ	3.5–37.2	164	2541.1	13.7 at 33 GHz	FR-4
^66^	0.256 λ × 0.247 λ	2.75–71	185	2912.2	12 at 60 GHz	Rogers RT5870
^67^	0.17 λ × 0.13 λ	0.95–13.8	173	7871.4	6 at 13.8 GHz	Rogers RT5880
^68^	0.35 λ × 0.23 λ	3–26	158	1962.7	7 at 26 GHz	FR-4
^69^	0.23 λ × 0.25 λ	3.8–68	179	3015.0	13 at 48 GHz	FR-4
^70^	0.19 λ × 0.31 λ	3–60	181	3073.0	12 at 30 GHz	Rogers RT5880
^71^	1.148 λ × 1.148 λ	17.22–180	165	125.2	10 at 110 GHz	FR-4
^72^	0.28 λ × 0.285 λ	1.4–20	173	2178.0	8 at 8.5 GHz	polyester nonwoven fabric
^73^	0.33 λ × 0.36 λ	2–30	175	1473.0	Not reported	FR-4
^41^	0.17 λ × 0.37 λ	14–18.8	172	2762.7	7 at 11 GHz	FR-4
^74^	0.97 λ × 0.8 λ	5–7	33.33	43.23	8.9	Rogers RT5880
^75^	0.16 λ × 0.29 λ	2.9–10.7	115	2406.9	5.3 at 8.5 GHz	FR-4
^24^	0.38 λ × 0.55 λ	3–35	168	805.84	6 at 13 GHz	FR-4
^37^	0.30 λ × 0.23 λ	2.2–22.1	163	2393.7	6.5 at 22 GHz	FR-4
^76^	0.18 λ × 0.33 λ	2.4–24.3	164	2718.1	4.4 at 12 GHz	FR-4
^77^	0.29 λ × 0.29 λ	2.9–18	144	1718.2	4.2 at 15 GHz	FR-4
^78^	0.22 λ × 0.24 λ	3–11.2	115	2187.4	5.4 at 9.8 GHz	FR-4
^79^	0.239 λ × 0.2397 λ	2.75–28	164	2871	4.80	FR-4
^80^	0.32 λ × 0.40 λ	1.2–15	170.3	1330.46	7	FR-4
^81^	0.326 λ × 0.28 λ	2.8–40	173.8	1904	7..16	FR-4
^82^	0.43 λ × 0.481 λ	4.37–16	114	551.77	2	Rogers RT5880
^83^	0.27 λ × 0.32 λ	2.88–14	131.17	1424.88	7.8 at 12.39 GHz	FR-4
^84^	0.21 λ × 0.15 λ	2.34–20	158.10	5019.11	5.25	FR-4
^85^	0.16 λ × 0.18 λ	1.25–40	188	6523	9.7	
^86^	0.33 λ × 0.33 λ	2.3–40	178	1732	5.81	FR-4
^87^	0.5 λ × 0.5 λ	2.9–30	165	2894	6.2	FR-4
^88^	0.34 λ × 0.36 λ	1.68–26	175.72	1435.6	5.5	FR-4
^89^	0.3 λ × 0.3 λ	3–20	147	1633	6	FR-4
^90^	0.56 λ × 0.56 λ	2.9–40	172	2897	13.5	FR-4
^91^	0.27 λ × 0.27 λ	2.3–34.8	175.2	2403.3	7.21	FR-4
^92^	0.188 λ × 0.138 λ	1.66–56.1	195	7516	7	PET paper ${\varepsilon }_{r}=3.2$ $tan\delta =0.022$
^93^	0.34 λ × 0.34 λ	2.59–31.14	169	1461.9	5	Rogers RT5880
^94^	0.25 λ × 0.20 λ	3.035–17.39	140.56	2800	4.56	FR-4
^95^	0.37 λ × 0.23 λ	4–40	164	1927	8	FR-4
^96^	0.21 λ × 0.18 λ	2.47–13	130.7	3457.67	–	FR-4
^97^	0.42 λ × 0.5 λ	3–10.6	111.7	531.90	5.2	FR-4
^98^	0.30 λ × 0.23 λ	3.68–31.61	158	2289	9.78	FR-4
Proposed	0.26 λ × 0.28 λ	2.9–29.2	164	2450.0	8.1 at 27 GHz	Transparent Plexi-glass

23 $${\lambda }_{l}=\frac{{L}_{g}}{{\lambda }_{0}} and {\lambda }_{w}=\frac{{W}_{g}}{{\lambda }_{0}}$$

Here ${W}_{g}$ and ${L}_{g}$ are the electrical width and length of the ground plane, respectively, and λ0 is the cut of wavelength which is the ratio of speed of light ‘c’ and the lower edge frequency (${f}_{l}$,), and BW present indicates the bandwidth in percent which is calculated according to the following equation which in relation 20, ${f}_{high}$ and ${f}_{low}$ show lower and higher working frequency bands, respectively. The larger the BDR value, the smaller the designed antenna and the larger the bandwidth.

## Conclusion

In this paper a transparent antenna with CPW structure was designed and verified for ultra-band width application and fine tuning parameters for impedance bandwidth expansion is implemented. A ground plane and radiation patch with several cuts were used for the sake of antenna transparency and impedance bandwidth. The proposed structure for the antenna reduces to BDR 2450 which represents a comparatively high BDR in comparison with the previously proposed structures. This fact confirms the wide bandwidth and compactness of the antenna. The proposed structure guarantees that the simulated frequency ranges from 2.9 to 29.2 GHz The measured frequency range of the produced sample antenna is matched with the simulated sample. The interest of the antenna ranges from 1 to 8.1 dB. The antenna efficiency is overall the working frequencies. The antennas specification is verified and checked with and without solar cells. This verification shows that solar panels have a tiny negative impact on antennas gain and have a huge positive effect on antennas bandwidth. Electrical dimensions, operating bandwidth (BW), bandwidth ratio (BWR), gain peak and beneath antenna substance were compared with the previous designs and schemas. We observed that the proposed antenna is performing optimally in comparison with other designs. Furthermore, to ensure that antenna is performing optimally we have tested and checked the antenna using a MIMO schema. At this step all of the vital parameters of the MIMO antenna such as electrical correlation coefficient (EEC), mean effective gain, diversity gain, total active reflection and channel capacity loss (CCL) is checked and the acquired appropriate/optimal values verify the optimal performance of the antenna in MIMO form. Due to these advantages, the designed antenna is applicable in wireless communication systems such as L, S, C, X, Lca, K, Ka and Q. In this article, transparent plexi-glass is used as a transparent substrate. The use of a transparent substrate, which has made the antenna transparent, and this feature has made it possible to use solar cells in the antenna. In addition to improving the performance of the antenna and the main characteristics of the antenna, the use of Plexiglas substrate has reduced construction costs by 10 times compared to other substrates. This substrate can be introduced as a cheap substrate with high resistance and high transparency. In previous papers, for the construction of the radiation part, metal oxides were used which, could interfere with the antenna radiation function. In this antenna, a layer of copper sheet that is precisely cut by a CNC laser is used. This antenna can be used in satellites with solar cells. It can be applied in vehicles glasses, Wireless CCTV cameras and wherever there is a need for an electrical power supply beside an antenna. The wideband transparent antenna has potential applications across various sectors including remote energy supply, wearable technology, and solar energy recovery. A plausible application for implementing a super wideband transparent antenna on a solar cell for self-driving cars is to enhance the vehicle's connectivity and communication systems. with regards to wearable technology, embedding the transparent antenna in clothing or other wearable devices could facilitate seamless connectivity without the need for bulky wires or antennas. This would be particularly useful in remote areas where traditional power sources are not available. Additionally, the transparency of the antenna would allow more light to pass through to the solar cell, further increasing its efficiency. This technology could be used in various applications such as in space exploration, where solar power is often used as a primary source of energy (Supplementary Information).

## Supplementary Information

Supplementary Information.

## Data Availability

The datasets generated during and/or analysed during the current study are available from the corresponding author on reasonable request.
